# Blood pressure fluctuation and hypertension in patients with Parkinson's disease

**DOI:** 10.1002/brb3.179

**Published:** 2013-10-11

**Authors:** Tetsuro Tsukamoto, Yoshimi Kitano, Sadako Kuno

**Affiliations:** 1 Department of Neurology, Numazu Rehabilitation Hospital2510-22 Kamikanuki-mandagahara, Numazu, Shizuoka-ken, 410-0813, Japan; 2 Department of Laboratory, Numazu Rehabilitation Hospital2510-22 Kamikanuki-mandagahara, Numazu, Shizuoka-ken, 410-0813, Japan; 3 Department of Neurology, Kyoto Shijo Hospital272-6 Shijohorikawamachi, Higashihorikawadori-shijoukudaru, Shimokyoku, Kyoto, 600-0000, Japan

**Keywords:** 24-h ABPM, autonomic dysfunction, fluctuating blood pressure, hypertension, Parkinson's disease

## Abstract

**Objectives:**

Blood pressure (BP) abnormalities have been known in Parkinson's disease (PD) patients. The present study aimed at determining how the BPs of PD patients fluctuate in a day.

**Methods:**

A total of 37 PD patients and 44 OD (other disease) patients, all of who were inpatients, were monitored every 30 min by 24-h ambulatory blood pressure monitoring (ABPM).

**Results:**

The average systolic BP and the number of patients who showed postprandial hypotension were not different between the two groups. However, occurrence of nocturnal hypertension, BP fluctuation of over 100 mmHg in a day and BP of over 200 mmHg were significantly more frequently observed in the PD patients than in the OD patients. In the PD patients, these parameters were not different between those who were suffering from the disease for less than 10 years and those with the disease for 10 years or longer, as well as between those who had a Hoehn–Yahr staging scale of 2–3 and those with a scale of 4–5.

**Conclusion:**

Twenty-four-hour ABPM, not BP measurement once a day, enables us to determine the actual BP in PD patients. Although hypotension is a severe risk factor for falling and syncope, we emphasize the importance of monitoring rather hypertension and fluctuating BP in PD patients that may lead to a variety of other undesirable conditions. Management of hypotension, hypertension, and BP fluctuation is an important issue in the future.

## Introduction

In addition to the motor dysfunctions caused by Parkinson's disease (PD) (e.g., resting tremor, muscle rigidity, bradykinesia, and postural instability), nonmotor dysfunctions such as psychiatric symptoms, dementia, sleep disorders, pains, and autonomic dysfunctions have recently been recognized (Ziemssen and Reichmann 2010; Jain 2011). Among various autonomic dysfunctions in PD, blood pressure (BP) abnormalities such as orthostatic hypotension (Gross et al. 1972; Goldstein et al. 2005; Ziemssen and Reichmann 2010; Sharabi and Goldstein 2011), postprandial hypotension (Ejaz et al. 2006; Luciano et al. 2010), and nocturnal hypertension (Ejaz et al. 2006; Ziemssen and Reichmann 2010; Sharabi and Goldstein 2011; Sommer et al. 2011) are known. However, medical personnel working in hospitals, nursing homes, visiting nursing, or group services often identify extreme BP fluctuations in PD patients and are troubled by excessively high or low BP which occasionally accompanies syncope, while most patients rarely complain of any symptom associated with such abnormal BP (Stuebner et al. 2013). In order to determine how the BPs of PD patients fluctuate in a day, we performed 24-h ambulatory blood pressure monitoring (ABPM) (Mallion et al. 1999).

With regard to the BP abnormalities in PD patients, there are several published studies that principally emphasize the importance of monitoring nocturnal hypertension, postprandial hypotension, and orthostatic hypotension (Senard et al. 1992; Ejaz et al. 2006; Schmidt et al. 2009; Stuebner et al. 2013). The present study demonstrates that the BPs of the PD patients fluctuate greatly in the range of 100 mmHg in a day, occasionally exceeding 200 mmHg.

The PD patients in this study included those who were having Parkinson's disease (PD), Parkinson's disease with dementia (PDD), or dementia with Lewy bodies (DLB). All the patients with PDD or DLB began with PD and had been treated for a long period as PD, and later advanced to have cognitive impairment, or fluctuating consciousness or psychosis such as hallucination and delusion. No patient with DLB began with dementia in this study. Recently PD, PDD, and DLB are each considered to be synucleinopathy as a disease entity (van den Berge et al. 2012; Kitao et al. 2013; Kubo and Hattori 2013).

The ABPM was performed in the hospitalized condition. In a home setting, BP is influenced by various daily stimuli and, therefore, the assessment of BP fluctuation may be more difficult.

## Methods

ABPM was performed every 30 min for 24 h using TM-2431 (A & D Company, Limited, Tokyo, Japan). A laboratory technical officer mounted a cuff on an upper arm of the respective patients and BP was recorded automatically. The daily activities of the patients such as rising, going to bed, taking meals, and exercising were recorded by attending nurses.

Examined were 37 PD patients including those who were having PD, PDD, or DLB. All the patients with PDD or DLB began with PD as mentioned above. The average Hoehn–Yahr staging scale of the PD patients was 3.9 (2–5). Also examined were 44 patients with other diseases (OD) such as cerebrovascular disease, femoral neck fracture, myasthenia gravis, and Guillain–Barre syndrome, who were not healthy and hospitalized for rehabilitation. All the patients examined were inpatients and were selected because they had no acute illness. The control subjects had various diseases and some of them could have autonomic dysfunction. They were transferred to our hospital for rehabilitation after having been treated at the respective previous hospitals and were already in a chronic and stable state. They did not complain of any symptom related to BP abnormality.

The average age of the patients with PD and that of the patients with OD were 75.2 (46–91) and 72.6 (39–85), respectively. Further, the gender ratio of these two groups (male:female) was 18:19 and 20:24, respectively (Table 1).

**Table 1:** The number of patients, gender ratio, average age, Hoehn–Yahr staging scale (H-Y), average systolic BP, and the standard deviation (SD) of the systolic BP

Disease	No. of patients	Gender (M/F)	Age	H-Y	Average systolic BP ± SD
PD	37	18/19	75.2 (46–91)	3.9 (2–5)	133.7 ± 18.8 mmHg
OD	44	20/24	72.6 (39–89)		129.1 ± 14.8 mmHg
*P*					0.234

PD, Parkinson's disease; OD, other diseases.

Nocturnal hypertension was defined as a condition where a nocturnal supine BP (from 7 pm to 6 am) was higher than a daytime BP. Postprandial hypotension was defined as a condition where a systolic BP was lower than 20 mmHg within 90 min after the beginning of a meal that was observed at least twice in three meals. The patients with percutaneous endoscopic gastrostomy (nine patients with PD and one patient with OD) were excluded for assessing postprandial hypotension. A ΔBP of over 100 mmHg (ΔBP > 100 mmHg) was defined as a condition where the systolic BP fluctuation was greater than 100 mmHg in a given period of 24 h.

Statistical analyses were performed by using Welch's *t* test and Fisher's exact probability test.

## Results

Nocturnal hypertension was observed in 64.9% of the patients with PD and 18.2% of the patients with OD. Postprandial hypotension was observed in 71.4% of the patients with PD and 51.2% of the patients with OD. A BP fluctuation of over 100 mmHg (ΔBP > 100 mmHg) was observed in 67.6% of the patients with PD, but only in 13.6% of the patients with OD. A BP of over 200 mmHg (BP > 200 mmHg) was observed in a period of 1 day in 35.1% of the patients with PD and 13.6% of the patients with OD.

The statistical analysis with Welch's *t* test showed no significant difference in the average BPs between the two groups, but the highest systolic BP during the monitoring was higher in the PD patients (average ± standard deviation = 194 ± 23 mmHg) than in the OD patients (177 ± 24 mmHg) (*P* < 0.05) and the lowest systolic BP was lower in the patients with PD (89 ± 14 mmHg) than in the patients with OD (97 ± 15 mmHg) (*P* < 0.05). Furthermore, Fisher's exact probability test demonstrated that nocturnal hypertension (*P* < 0.001), ΔBP > 100 mmHg (*P* < 0.001), and BP > 200 mmHg (*P* < 0.05) were observed significantly more often in the patients with PD than in the patients with OD. There was no significant difference between the two groups of patients in terms of postprandial hypotension, although the patients with PD tended to develop postprandial hypotension more often (71.4%) than the patients with OD (51.2%) (Tables 1 and 2).

**Table 2:** BP fluctuation, hypertension of over 200 mmHg, nocturnal hypertension, and postprandial hypotension in PD patients and OD patients

Diseases	ΔBP > 100 mmHg	BP > 200 mmHg	NH	PH
PD	24/37 (64.9%)	13/37 (35.1%)	24/37 (64.9%)	20/28 (71.4%)
OD	6/44 (13.6%)	6/44 (13.6%)	8/44 (18.2%)	22/43 (51.2%)
*P*	<0.001	<0.05	<0.001	0.073

ΔBP > 100 mmHg, systolic BP fluctuation of over 100 mmHg in 24 h; BP > 200 mmHg, systolic BP of over 200 mmHg in 24 h; NH, nocturnal hypertension; PH, postprandial hypotension.

In the PD patients, these parameters were not different between those who were suffering from the disease for less than 10 years and those with the disease for 10 years or longer, as well as between those who had a Hoehn–Yahr staging scale of 2–3 and those with a scale of 4–5 (Tables 3 and 4).

**Table 3:** BP data of PD patients who were suffering from the diseases for less than 10 years or for 10 years or longer

Duration	No. of patients	Average systolic BP ± SD	ΔBP > 100 mmHg	BP > 200 mmHg
Less than 9 years	19 (51.4%)	137.7 ± 21.9	14/19 (73.7%)	8/19 (42.1%)
10 years or longer	18 (48.6%)	128.9 ± 13.9	10/18 (55.6%)	5/18 (27.8%)
*P*		0.153	0.209	0.286

**Table 4:** BP data of PD patients who had a Hoehn–Yahr scale (H-Y) of 2–3 or 4–5

H-Y scale	No. of patients	Average systolic BP ± SD	ΔBP > 100 mmHg	BP > 200 mmHg
H-Y 2–3	13 (35.1%)	128.8 ± 12.8	7/13 (53.8%)	4/13 (30.8%)
H-Y 4–5	24 (64.9%)	136 ± 21.1	17/24 (70.8%)	9/24 (37.5%)
*P*		0.204	0.687	0.485

During the examination, no patient developed syncope, dizziness, or any other symptoms related to the BP change.

Prescribed drugs for the patients with PD were l-DOPA, dopamine agonists, selegiline, entacapon, zonisamide, and/or L-threo-DOPS. No patient received fludrocortisone. The relationships between the BP and the respective drugs prescribed for PD, however, were not clear due to the small number of the examined patients and occurrence of unpredictable BP fluctuation.

## Discussion

As conventionally known, the PD patients exhibited a tendency to develop orthostatic hypotension (Gross et al. 1972; Goldstein et al. 2005; Ziemssen and Reichmann 2010; Sharabi and Goldstein 2011), postprandial hypotension (Ejaz et al. 2006; Luciano et al. 2010), and nocturnal hypertension (Ejaz et al. 2006; Ziemssen and Reichmann 2010; Sharabi and Goldstein 2011; Sommer et al. 2011). Although orthostatic hypotension may be a risk factor leading to dizziness, syncope, and falling, many patients are known to be asymptomatic (Stuebner et al. 2013). Importantly, in the present study, the PD patients were found to experience considerable intraday BP fluctuation, and there were observed many cases where the fluctuation was larger than 100 mmHg in terms of the difference between the highest and the lowest systolic blood pressures. Furthermore, although the average BP of the PD patients was not significantly different from that of the control patients, the highest systolic BP during the monitoring was higher in the PD patients than in the OD patients and the lowest systolic BP was lower in the PD patients than in the OD patients, suggesting that the PD patients experience greater BP fluctuations. Such larger BP fluctuations may confuse attending medical personnel who happen to notice a high or low abnormal BP in patients. In addition, it is intriguing that some of the patients in the advanced stage lying in bed all day long also showed large BP fluctuations, suggesting that the cardiovascular autonomic function is severely impaired and BP regulation is lost in these patients.

In the treatment of PD, conventionally, occurrence of low BP has been regarded as a problem (Ziemssen and Reichmann 2010; Jain 2011; Sharabi and Goldstein 2011); however, the present study found that the PD patients frequently experience a high BP of 200 mmHg or higher, which indicates that they may potentially be subjected to risks of high BP several times a day. Such a condition may go unnoticed over a prolonged period of time because mere routine BP measurement performed once a day or so is unable to determine their exact BP changes (Mallion et al. 1999; White et al. 2011). Ziemssen and Reichmann (2010) provide an example of ABPM in a PD patient, which also shows BP fluctuations and occurrence of a high BP of over 200 mmHg during night. A prominent BP fluctuation accompanying hypertension may potentially induce cerebral stroke, cardiovascular disorder, and/or organopathy; therefore, it is rather required to select a drug capable of stabilizing the BP (Parati and Mancia 2001; Brickman et al. 2010).

In terms of the average BP, ΔBP > 100 mmHg, and BP > 200 mmHg, there was no significant difference between the PD patients who were suffering from the disease for less than 10 years and those with the disease for 10 years or longer as well as between those who had a Hoehn–Yahr staging scale of 2–3 and those with a scale of 4–5. This suggests that the autonomic dysfunction may even begin in the early stage of the disease (Asahina et al. 2013; Stuebner et al. 2013); however, as this study was performed only for inpatients whose disease conditions had fairly advanced and the sample size was small, it is yet to be determined as to how the BP fluctuates in an earlier stage of the illness. Furthermore, the reason why abnormal BP fluctuations were frequently observed even in the control subjects is speculated to be because they were inpatients and aged (Haensch and Jorg 2005; Stuebner et al. 2013), that is, not completely healthy individuals who were suffering from cerebrovascular disease and the like. As the control group, the use of healthy controls would have been better suited for evaluating the difference between the disease and the health, and if healthy controls were assessed, the difference could have been more prominent and more accurately identified, but it is not practical to gather aged healthy individuals and evaluate them in the hospital. Furthermore, most aged individuals may already have some diseases and have autonomic dysfunction to some extent (Haensch and Jorg 2005; Stuebner et al. 2013).

In conclusion, we emphasize that rather hypertension and fluctuating BP, which may lead to a variety of other undesirable conditions (Parati and Mancia 2001; Brickman et al. 2010), should be monitored in PD patients, even though hypotension in PD is a severe risk factor for falling and syncope. Management of hypotension, hypertension, and BP fluctuation is an important issue in the future.
